# Lysine-specific histone demethylase 1 inhibition promotes reprogramming by facilitating the expression of exogenous transcriptional factors and metabolic switch

**DOI:** 10.1038/srep30903

**Published:** 2016-08-02

**Authors:** Hao Sun, Lining Liang, Yuan Li, Chengqian Feng, Lingyu Li, Yixin Zhang, Songwei He, Duanqing Pei, Yunqian Guo, Hui Zheng

**Affiliations:** 1 CAS Key Laboratory of Regenerative Biology, Guangdong Provincial Key Laboratory of Stem Cell and Regenerative Medicine, Guangzhou Institutes of Biomedicine and Health, Chinese Academy of Sciences, Guangzhou 510530, China; 2 College of Biological Sciences and Technology, Beijing Forestry University, Beijing 100083, China

## Abstract

Lysine-specific histone demethylase 1 (LSD1) regulates histone methylation and influences the epigenetic state of cells during the generation of induced pluripotent stem cells (iPSCs). Here we reported that LSD1 inhibition via shRNA or specific inhibitor, tranylcypromine, promoted reprogramming at early stage via two mechanisms. At early stage of reprogramming, LSD1 inhibition increased the retrovirus-mediated exogenous expression of *Oct4, Klf4*, and *Sox2* by blocking related H3K4 demethylation. Since LSD1 inhibition still promoted reprogramming even when iPSCs were induced with small-molecule compounds in a virus-free system, additional mechanisms should be involved. When RNA-seq was used for analysis, it was found that LSD1 inhibition reversed some gene expression changes induced by OKS, which subsequently promoted reprogramming. For example, by partially rescuing the decreased expression of *Hif1α,* LSD1 inhibition reversed the up-regulation of genes in oxidative phosphorylation pathway and the down-regulation of genes in glycolysis pathway. Such effects facilitated the metabolic switch from oxidative phosphorylation to glycolysis and subsequently promoted iPSCs induction. In addition, LSD1 inhibition also promoted the conversion from pre-iPSCs to iPSCs by facilitating the similar metabolic switch. Therefore, LSD1 inhibition promotes reprogramming by facilitating the expression of exogenous transcriptional factors and metabolic switch.

Lysine-specific demethylase 1 (*Lsd1/Kdm1a/Aof2*) functions through a FAD-dependent oxidative reaction to specifically catalyze the demethylation of H3K4me1/2 or H3K9me1/2^1^,^2^. LSD1 induces transcriptional repression via H3K4 demethylation when it forms complexes with the CoREST or NuRD co-repressors, and induces transcriptional activation via demethylation of the inhibitory H3K9 when complexes with androgen or estrogen nuclear receptors^2^. It has been reported that the phosphorylation on H3T6 by protein kinase C switches the demethylation activity of LSD1 from H3K4me1/2 to H3K9me1/2^3^. However, how LSD1 regulates transcription remains unclear because of its complex interactions.

LSD1 also has several non-histone substrates. LSD1 removes methylation at K370 of p53 which regulates the association of p53 with p53-binding protein 1 and so modulates p53 activity^4^. In addition, deletion of *LSD1* in embryonic stem cells (ESCs) results in higher methylation of DNMT1, reduced stability of DNMT1 and consequently decreased global levels of DNA methylation^5^. LSD1 also affects DNA damage-induced cell death through modifications on E2F1, and affects the cell cycle via MYPT1 demethylation^6^,^7^.

LSD1 plays critical functions during embryonic development and the differentiation of ESCs. *LSD1* deletion in mice leads to embryonic lethality at approximately day 6 due to an aberrant developmental program^5^,^8^. LSD1 regulates the balance between self-renew and differentiation by silencing several developmental genes with bivalent domains^9^,^10^. Since LSD1 occupies the enhancer and core promoters of some Oct4/Sox/Nanog-regulated targets^10^, it is reasonable to propose interactions between LSD1 and pluripotency-related transcriptional factors (TFs). Both Oct4 and Nanog interact with LSD1 or the LSD1-NuRD complex^11^,^12^, and their specific interaction could form a bridge between pluripotent transcription factors and LSD1.

Because epigenetic regulation plays critical roles during the generation of induced pluripotent stem cells (iPSCs)^13^,^14^, LSD1 has also been studied extensively in this process. Lithium greatly enhances iPSCs generation via down-regulation of *LSD1*^15^. In addition, an LSD1 inhibitor is included in the cocktail of small-molecule compounds which induces mouse somatic cells to iPSCs (CiPSCs)^16^. However, opposite observations also exist, LSD1 inhibition leads to an increase of *mTOR* expression, which subsequently impairs the reprogramming^17^. As a subunit of the larger LSD1 complex, the co-repressor Rcor2 can substitute for Sox2 during reprogramming, which is consistent with the critical functions of Sox2 in regulating the sensitivity of cancer cells to LSD1 inhibition^18^,^19^.

The beneficial roles of LSD1 inhibition were further confirmed recently during a shRNA library screening with the hTERT-stabilized secondary fibroblast reprogramming system and during a C/EBPα-accelerated B cell reprogramming with four Yamanaka factors^20^,^21^. In addition, the ability of LSD1 to promote oxidative metabolism of white adipose tissue suggested its potential involvement in the metabolic switch from oxidative to highly glycolytic at early stage of reprogramming^22^,^23^. Therefore, we aimed to further explore the functions of LSD1 during iPSC generation by determining the time-dependent regulatory roles of LSD1 inhibition, identifying the key effectors down-stream the inhibition, and demonstrating the potential influences on the metabolic switch from oxidative to glycolytic.

## Results

### LSD1 inhibition promotes iPSCs generation

**Figure 1:**
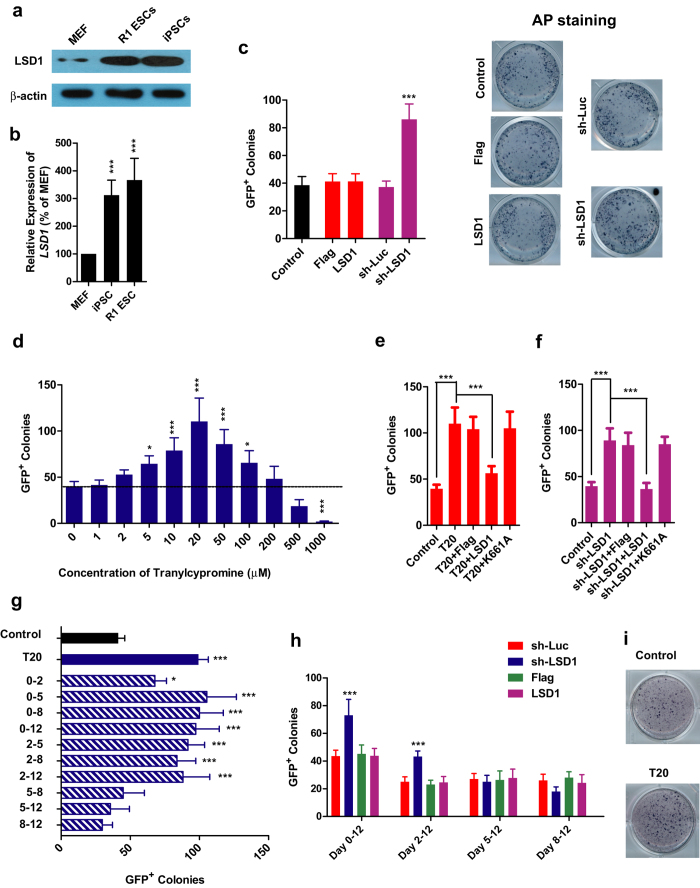
LSD1 inhibition promotes iPSCs generation. (**a,b**) *Lsd1* expression was determined with immunoblotting ((**a**), n = 5) and qPCR ((**b**), n = 5) in MEFs, iPSCs and R1 ESCs. (**c**) Reprogramming was performed with *Lsd1* up- and down-regulation. GFP^+^ colonies numbers and AP staining results were summarized (n = 6). (**d**) Different concentrations of LSD1 inhibitor, tranylcypromine, were used for reprogramming. Efficiencies were evaluated by counting GFP^+^ colonies (n = 6). (**e,f**) 20 μM tranylcypromine (T20), retrovirus encoding Flag vector, LSD1, LSD1 deficient mutant, K661A, and shRNA against *Lsd1* (sh-LSD1) were used for reprogramming as indicated. GFP^+^ colonies were counted (n = 6). (**g**) T20 was used during indicated periods during reprogramming, and GFP^+^ colonies were counted (n = 6). (**h**) Retrovirus encoding Flag vector, *Lsd1*, shRNA against *Luciferese* (sh-Luc) and sh-LSD1 were delivered at different time points during reprogramming before counting GFP^+^ colonies on Day 12 (n = 6). (**i**) T20 was used for HFFs reprogramming. AP staining was performed to determining the efficiencies (n = 5). One-way *ANOVA* with Dunnett’s test as a post-hoc was used to compare the experimental with control groups. The gels used for different antibodies were run under the same experimental conditions (**a**). Representative images were cropped from the original images with no modification on the relative intensities. The full length blots were provided in Supplementary Figure S8.

Since *Lsd1* expression in iPSCs and R1 ESCs were significantly higher than that in MEFs (Fig. 1a,b), a possible beneficial role of LSD1 for reprogramming was suggested. However, retrovirus-mediated over-expression of *Lsd1* did not affect the efficiency of reprogramming (Fig. 1c, Supplemental Fig. S1). In contrast, down-regulating *Lsd1* with shRNA promoted iPSCs generation significantly, to more than 2 folds of control level (Fig. 1c, Supplemental Fig. S1).

To determine the functions of LSD1 during reprogramming in detail, a LSD1 inhibitor, tranylcypromine with an IC_50_ of less than 2 μM^24^, was used. Tranylcypromine had a variable effect on reprogramming efficiency, increasing the number of GFP^+^ colonies with increasing concentrations, but declining at higher levels possibly due to cell toxicity or off-target effects (Fig. 1d). The optimal concentration was 20 μM (T20, about 10 folds of IC_50_) which increased reprogramming efficiency to around 3 folds of the control level (Fig. 1d). In addition, LSD1 but not its mutant, K661A which has little demethylase activity^25^, impaired T20- or sh-LSD1-induced increases in reprogramming efficiencies (Fig. 1e,f). These observations not only confirmed the effects of tranylcypromine and shRNA were through LSD1, but also indicated the critical roles of LSD1’s demethylase activity in promoting iPSCs generation.

We next examined the time-dependent functions of LSD1 during reprogramming. The beneficial roles of LSD1 inhibition (T20 treatment) was only observed at early stage of reprogramming, from Day 2 to 5 during a 12-day reprogramming (Fig. 1g). Similarly, shRNA against *LSD1* only promoted reprogramming significantly at early stage though its suppression last until late stage of reprograming (Fig. 1h, Supplemental Fig. S1).

Since c-Myc is also frequently used for reprogramming^14^, the effects of LSD1 inhibition in a reprogramming system with *Oct4, Klf4, c-Myc* and *Sox2* (OKMS) was also determined. As indicated in Supplemental Fig. S1, LSD1 inhibition promoted reprogramming at early stage.

In addition, another inhibitor of LSD1, phenelzine also enhanced reprogramming with or without *c-Myc* in a dose-dependent manner (Supplemental Fig. S1). To further exclude the possible off-target effects, GSK2879552, a potent, selective, and irreversible LSD1 inhibitor^26^, was also determined to facilitate reprogramming with or without *c-Myc* in a dose-dependent manner (Supplemental Fig. S1). In addition, the beneficial effects of both phenelzine and GSK2879552 were limited to the early stage of reprogramming.

Furthermore, T20 also promoted the reprogramming of human foreskin fibroblasts as indicated by AP staining (Fig. 1i). These consistent results generated from different reprogramming systems further supported the beneficial effects of LSD1 inhibition.

iPSCs colonies generated with OKS and T20 had normal karyotypes, proper demethylation on both Nanog and Oct4 promoters, and the expression of endogenous pluripotency markers such as Nanog and SSEA1 (Supplemental Fig. S2). These iPSCs were able to form chimeric mice with germ line transmission (Supplemental Fig. S2).

### T20 does not affect cell cycle and global DNA methylation

Since both p53 and DNMT1 are reported LSD1 substrates^4^,^5^, LSD1 inhibition may facilitate reprogramming by modulating p53 methylation and subsequent cell cycle or by modulating DNMT1 methylation and subsequent global DNA methylation. It is possible that the observed beneficial effects on reprogramming were due to the alterations in cell cycle or global DNA methylation because of their influences on iPSCs generation^27^,^28^. Although the methylation levels of p53 and DNMT1 were not determined because of the lack of specific antibody, the possible involvement of their demethylation under current paradigm was excluded by determining their down-stream effects, modulations on cell cycle and global DNA methylation. The similar proliferation rates and lengths of different cell cycle phases in cells treated with or without T20 suggested that LSD1 inhibition did not affect reprogramming via a pathway from methylated p53 to cell cycle (Supplemental Fig. S3). The unchanged global DNA methylation levels suggested the influences from LSD1 inhibition were not mediated by DNMT1 or global DNA methylation.

However, the unchanged down-stream effects such as cell cycle and global DNA methylation might be resulted from the changes on expression and methylation levels simultaneously. To exclude this possibility, the expression of *p53* and *Dnmt1* were determined. The expression of *p53* and *Dnmt1* were not affected by LSD1 inhibition, suggesting that the methylation levels of these two protein were unchanged from another aspect (Supplemental Fig. S3). In addition, Apoptosis also seems unaffected by T20 treatment as the percentage of TUNEL^+^ cells remained unchanged (Supplemental Fig. S3).

### T20 increases exogenous *OKS* expression by preventing H3K4 demethylation

LSD1 is essential in maintaining low H3K4 methylation on endogenous retrovirus elements and subsequently suppresses their expression in ESCs^29^. Thus the abilities of T20 to affect retrovirus-delivered exogenous expression of *Oct4, Klf4*, and *Sox2* were determined. Although retrovirus delivery of OKS increased their expression to different extents, LSD1 inhibition with T20 further boosted their expression to about three folds.

**Figure 2:**
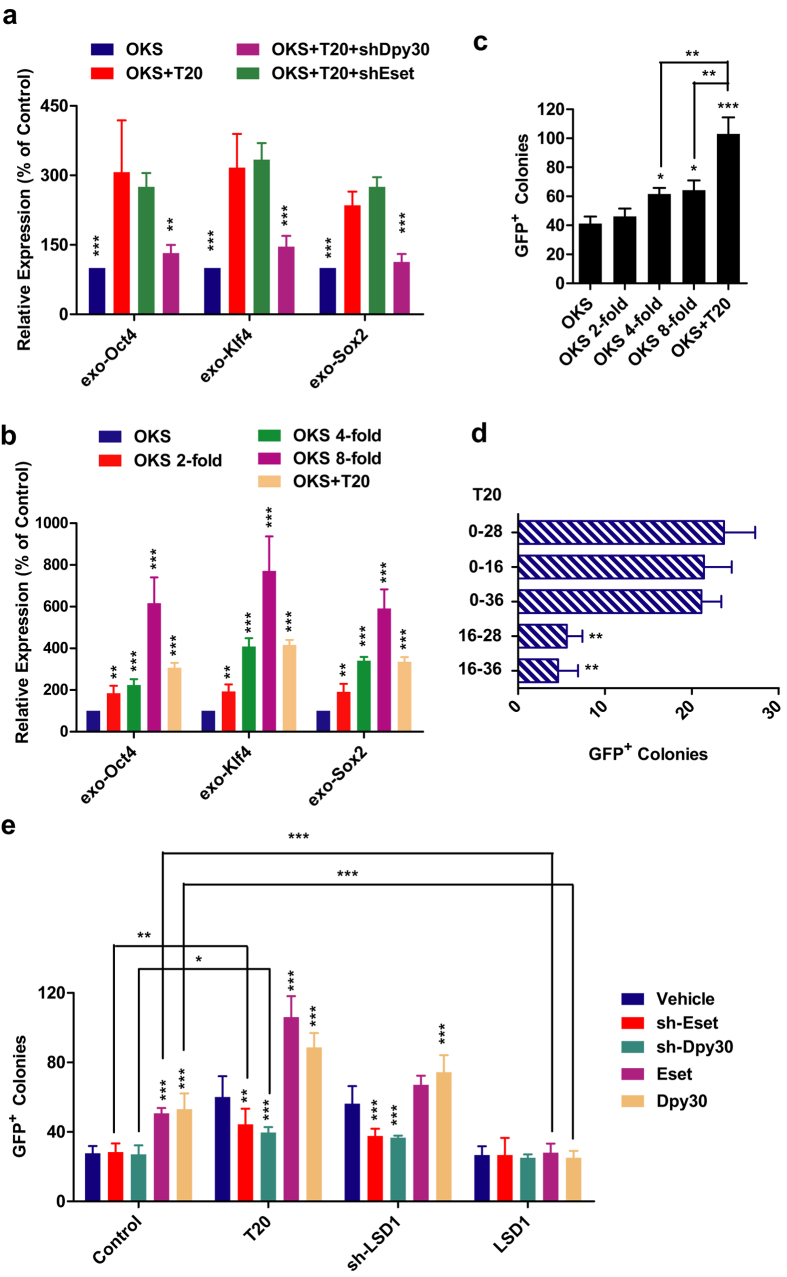
T20 promotes reprogramming by increasing exogenous expression of OKS. (**a**) Retrovirus encoding sh-Eset and sh-Dpy30 were used with T20 during iPSCs generation. Expression of exogenous *Oct4, Klf4* and *Sox2* were determined by qPCR (n = 5). (**b,c**) Different amounts of retrovirus were used for reprogramming. Exogenous expression of OKS were determined by qPCR on Day 3 during reprogramming and normalized to those in OKS group ((**b**), n = 5). GFP^+^ colonies numbers on Day 12 were summarized in ((**c**), n = 5). (**d**) CiPSCs were generated as reported previously and outlined in Methods. Tranylcypromine was used at 20 μM and in indicated time periods during reprogramming. GFP^+^ colonies were counted for efficiencies (n = 5). (**e**) Retrovirus encoding sh-Eset and sh-Dpy30 were used in different combinations with T20, retrovirus encoding sh-LSD1 and LSD1 for MEFs reprogramming. GFP^+^ colonies were counted to represent the efficiencies (n = 6). One-way ANOVA with Dunnett’s test as a post-hoc was used for (**a–d**), while Two-way *ANOVA* with Bonferroni’s test was used for (**e**).

To explore the connection between increased exogenous expression of OKS and LSD1-mediated histone demethylation, shRNAs against *Dpy30* and *Eset* were used (Supplemental Fig. S4). Since Dpy30 and Eset are critical for H3K4 and H3K9 methylation respectively^30^,^31^, blocking their expression should affect different parts of LSD1 inhibition. As indicated in Fig. 2a, increased exogenous expression of OKS could be blocked by *sh-Dpy30* but not *sh-Eset*. Therefore, LSD1 inhibition facilitates *OKS* expression by suppressing related H3K4 demethylation.

In order to determine the contributions of increased exogenous OKS expression to reprogramming, different amounts of viruses were used to infect MEFs at the beginning of reprogramming. The abilities of T20 to increase exogenous OKS expression were between those of 4-fold and 8-fold of viruses (Fig. 2b). Although both 4-fold and 8-fold of viruses increased iPSCs generation efficiency significantly, the reprogramming efficiencies induced by them were still lower than that of OKS+T20 (Fig. 2c), suggesting that increased OKS expression only accounted for part of T20’s effects.

To further confirm the observation above, the effects of T20 on reprogramming were determined in a virus-free system. When using the previous reported protocol to generate CiPSCs^16^, T20 also greatly promoted iPSCs generation at early stage (Fig. 2d). Therefore, LSD1 inhibition facilitates reprogramming via another pathway.

In addition, immunoblotting data suggested that T20 increased global levels of H3K4me2/3 and H3K9me2/3 (Supplemental Fig. S4). Furthermore, reducing the expression of *Dpy30* or *Eset* with shRNA significantly impaired but not fully blocked the beneficial roles of T20 (Fig. 2e). Therefore, LSD1 inhibition with T20 promotes iPSCs generation by regulating both H3K4 and H3K9 demethylation and through multiple pathways.

### T20 partially reverses gene expression changes induced by OKS

**Figure 3:**
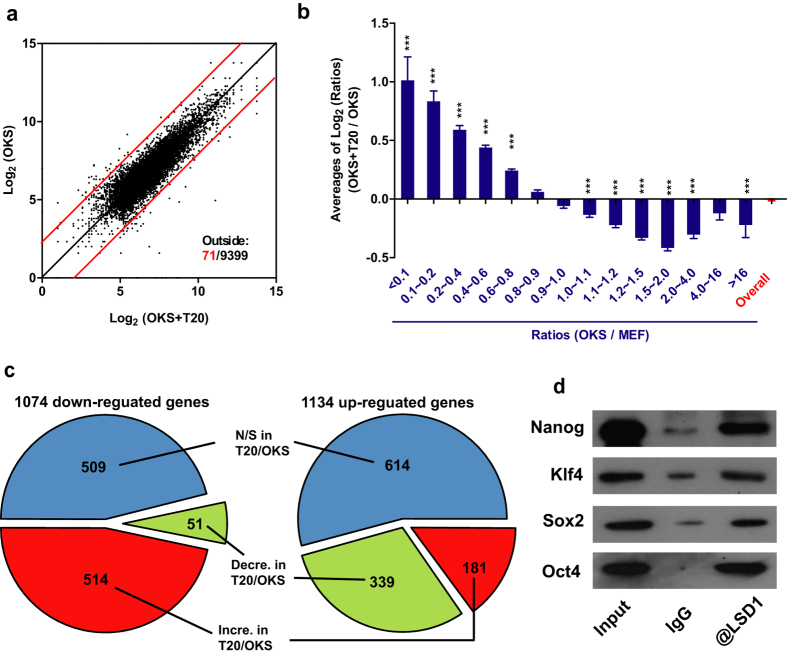
T20 reverses some expression changes induced by OKS. (**a**) Log_2_ values of RNA-seq readings on gene expression in OKS, and OKS+T20 groups were plotted against each other. Red lines were used to distinguish the genes with expression changes more than 5 folds. (**b**) 9399 genes identified in current RNA-seq were divided into 14 groups depending on expression ratios (OKS vs MEFs). The averages of ratios (OKS+T20 vs OKS, Log_2_ values) in these 14 groups were plotted. Stand errors were plotted. (**c**) 2208 genes with significant expression differences (over two folds) between MEF and OKS groups were analyzed separately, down-regulation in left panel and up-regulation in right. Within these genes, genes with expression ratios (OKS+T20 vs OKS) between 0.67 and 1.50 were classified as not significant (N/S, in Blue), genes with ratios over 1.50 were as increased (Incre. in Red), while genes with ratios below 0.67 were as decreased (Decre. in Green). Genes with increased expression were classified as reversed changes in left panel but as consistent changes in right panel. The classification of genes with decreased expression was just opposite. (**d**) *Oct4, Klf4, Sox2* and *Nanog* were over-expressed in MEFs via retrovirus system for three days, antibody against LSD1 was used for immuno-precipitation (n = 5). One-way ANOVA with Dunnett’s test as a post-hoc was used. The gels used for different antibodies were run under the same experimental conditions (**a**). Representative images were cropped from the original images with no modification on the relative intensities. The full length blots were provided in Supplementary Figure S8.

Since the beneficial roles of T20 on reprogramming was mostly at early stage, we performed RNA-seq analysis in MEFs and cells on Day 3 during reprogramming with (OKS+T20) and without T20 (OKS) (Supplemental Table S1). Gene expression profiles were compared between any two of these three samples, and totally three comparisons were performed (Fig. 3a, Supplementary Fig. S5). The difference between the expression profiles of OKS group and OKS+T20 group was smaller than the difference between MEF and OKS or between MEF and OKS+T20. Although the difference between OKS and OKS+T20 was the smallest in the three comparisons, it indicated the function of T20 and was focused for further analysis.

The 9399 genes with detectable expression in current RNA-seq were divided into 14 groups basing on their expression ratios, OKS vs MEF. It was found that OKS preferred to up-regulate genes with relatively low expression in MEFs, and down-regulate those with relatively high expression (Supplemental Fig. S5). This phenomenon was a typical pattern for gene expression changes. However, when compared the gene expression in OKS+T20 with those in OKS, T20 preferred to increase the expression of genes that were down-regulated by OKS, while to decrease those up-regulated by OKS (Fig. 3b). To be more accurate, within the 1074 genes that were down-regulated by more than 50% after OKS delivery, 509 genes had their expression changes un-affected by T20, 514 genes had their expression changes been significantly reversed, and only 51 genes were further down-regulated by T20 (Fig. 3c). Similarly, within the 1134 genes that were up-regulated by more than 100% after OKS delivery, 614 genes had their expression changes un-affected by T20, 339 genes had their expression changed been significantly reversed, and only 181 genes were further up-regulated by T20 (Fig. 3c). Therefore, T20 partially reverses gene expression changes induced by OKS. Since the expression profile of OKS+T20 group was still closer to OKS group than to MEF group, the ability of T20 to modulate gene expression could not overwhelmed those of Oct4, Klf4 and Sox2.

Considering the interactions between LSD1 and pluripotent transcriptional factors like Oct4, Sox2. Klf4 and Nanog (Fig. 3d), OKS might down-regulate genes with relatively high expression via H3K4 demethylation, while up-regulated those with low expression via H3K9 demethylation, with help from LSD1 at early stage of reprogramming. When LSD1 was inhibited, OKS-induced expression changes might be reversed.

### T20 assists in a metabolic switch during iPSCs generation

**Figure 4:**
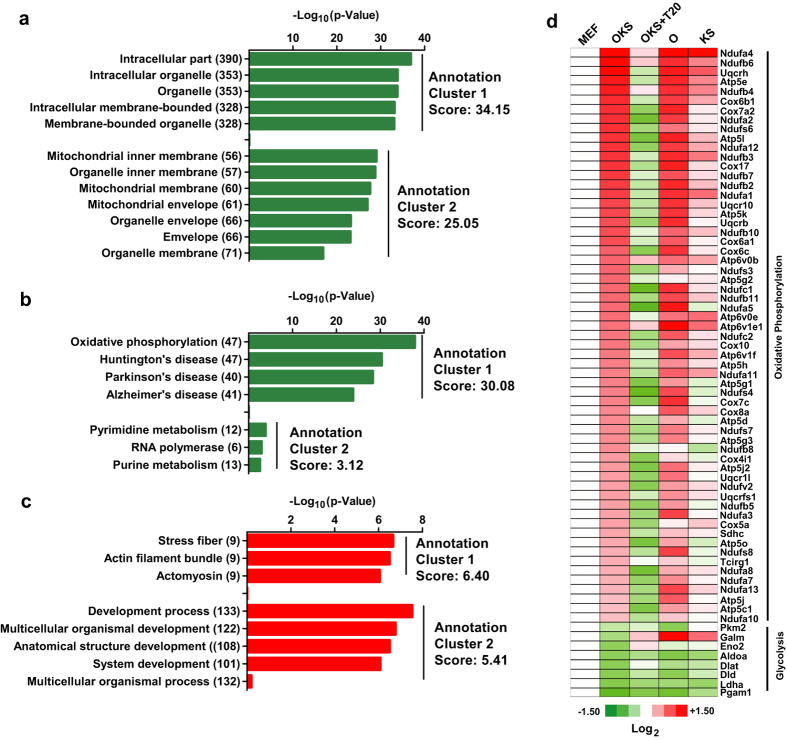
T20-down-regulated genes clusters in metabolic pathways. (**a–c**) Genes were significantly down-regulated by T20 were subjected for GO (**a**) and KEGG (**b**) analysis. Genes were significantly up-regulated by T20 were subjected for GO analysis (**c**). The top 2 clusters were listed. (**d**) 61 genes in OX pathway and 8 genes in glycolysis pathway which have significant differences between MEF and OKS groups and between OKS and OKS+T20 groups were listed.

To connect the abilities of T20 to reverse OKS-induced expression changes to its beneficial roles for reprogramming, genes with more than two-fold expression differences between OKS and OKS+T20 groups were analyzed with DAVID online service (http://david.abcc.ncifcrf.gov/) to identify the enriched Gene Ontology (GO) terms and Kyoto Encyclopedia of Genes and Genomes (KEGG) pathways^32^. Genes related to mitochondrial and oxidative phosphorylation pathway (OX, KEGG: 00190) were significantly enriched in T20-down-regulated genes (Fig. 4a,b, Supplemental Table S2), while genes related to stress fiber and development process were significantly enriched in T20-up-regulated genes (Fig. 4c, Supplemental Table S2).

Genes in OX pathway and another related pathway, glycolysis pathway (KEGG: 00010), were then analyzed basing on the information collected from KEGG website (http://www.genome.jp/kegg/). 98 out of 116 genes in OX pathway had detectable expression in the current RNA-seq. About 80% of these 98 genes were up-regulated by OKS on Day 3 during reprogramming and experienced down-regulation when T20 was included in reprogramming system simultaneously (Supplemental Table S3). On the other hand, 34 out of 60 genes in glycolysis pathway were detectable in the current RNA-seq. About 40% of these 34 genes were down-regulated by OKS on Day 3 and up-regulated when T20 was used (Supplemental Table S3). 61 OX genes and 8 glycolysis genes were further selected out because of the larger expression differences among MEF, OKS and OKS+T20 samples. Their expression levels were presented in Fig. 4c. Therefore, T20 reverses OKS-induced expression changes of genes in OX and glycolysis pathways.

**Figure 5:**
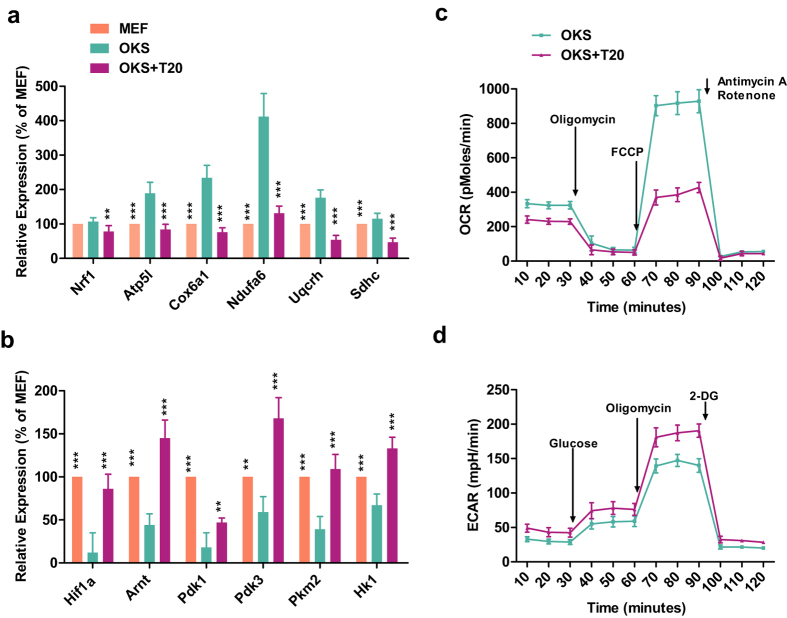
T20 helps the metabolic switch during reprogramming. (**a,b**) qPCR results of *Nrf1*, representative genes in OX pathway ((**a**), n = 5), *Hif1a*, and representative genes in glycolysis pathway ((**b**), n = 5) in MEFs, OKS and OKS+T20 groups were listed. (**c,d**) Seahorse XF24 extra-cellular flux analyzer (Seahorse Bioscience) was used to assess cellular energy metabolism on Day 3 during reprogramming with or without T20. Different inhibitor were added as indicated. OCR and ECAR results were presented in (**c,d**) respectively (n = 5). One-way ANOVA with Dunnett’s test as a post-hoc was used.

To confirm the RNA-seq results, qPCR was performed to determine the expression of several representative genes. Nuclear respiratory factor 1 (*Nrf1*) expression was not affected significantly by OKS or T20, but representative genes in the OX pathway were up-regulated by OKS and down-regulated by T20 (Fig. 5a). Hypoxia-inducible factor 1α (*HIF1α*) expression was down-regulated by OKS while reversed by T20, which was consistent with its down-stream glycolysis genes, *Pdk1, Pdk2*, and *Pkm2* (Fig. 5b). Seahorse XF24 extra-cellular flux analyzer (Seahorse Bioscience) was then used to assess cellular energy metabolism on Day 3 during reprogramming. According to the OCR and ECAR readings in Fig. 5c,d, T20 treatment shifted cells from oxidative to glycolytic metabolism. Since transition from oxidative to glycolytic metabolism is required during iPSC generation^33^, T20 promotes reprogramming by facilitating metabolic switch.

Similarly, shRNA against *Lsd1* and LSD1-specific inhibitor, GSK2879552, also decreased the expression of representative genes in the OX pathway, increased the expression of glycolysis genes, and facilitated the metabolic switch from oxidative to glycolytic when used together with *Oct4, Klf4* and *Sox2* during iPSCs generation (Supplementary Figure S6).

### T20 assists metabolic switch by modulating *Hif1a* expression

Interactions between LSD1 and NRF1 or HIF1α during metabolic regulation have already been reported^23^,^34^. In addition, when analyzing the −950 − +50 regions (related to the transcription start sites) of the 116 OX genes with Pscan online service (http://159.149.160.51/pscan/)^35^, binding motifs of transcription factors like NRF1, HINFP, GABPA, and HIF1α were enriched (Supplemental Table S4). Binding motifs of Klf4 and HIF1α were enriched in the corresponding regions of the 60 glycolysis genes. Thus NRF1 and HIF1α were selected for further studied. Since significant expression changes were only observed on *Hif1α* but not *Nrf1* under current paradigm (Fig. 5a,b), whether T20 facilitated metabolic switch by modulating *Hif1a* expression was further determined.

**Figure 6:**
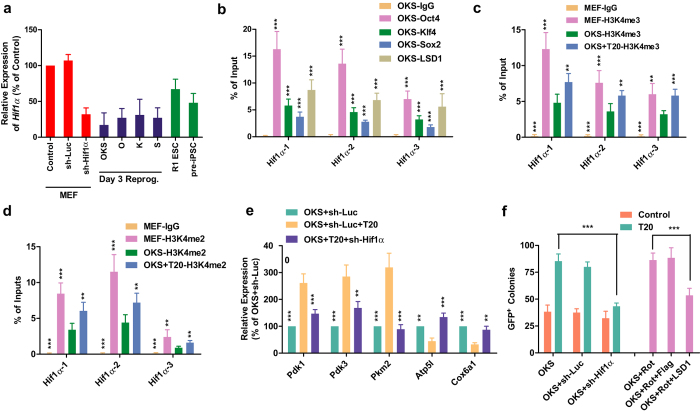
T20 facilitates the metabolic switch via Hif1α. (**a**) *Hif1a* expression was determined in MEFs, MEFs with sh-Luc or sh-Hif1α, ESCs, pre-iPSCs, and cells on Day 3 of reprogramming with OKS, O, K, or S (n = 5). (**b–d**) ChIP-qPCR with antibodies against Oct4, Klf4, Sox2, LSD1 (**b**), H3K4me3 (**c**) and H3K4me2 (**d**) were performed. Three sets of primers on *Hif1α* gene locus were used for qPCR. IgG was used as negative control and the results were normalized to the qPCR results from 1% inputs (n = 5). (**e**) qPCR results on genes in OX pathway and glycolysis pathway on Day 3 of reprogramming with OKS+sh-Luc, OKS+sh-Luc+T20, or OKS+sh-Hif1α+T20 (n = 5) (**f**) Retrovirus encoding sh-Hif1a was used with or without T20, and oxidative phosphorylation inhibitor, rotenone (Rot), was used with retrovirus encoding Flag or LSD1 during iPSCs generation. GFP^+^ colonies were counted (n = 6). One-way ANOVA with Dunnett’s test as a post-hoc was used.

Although *Hif1a* expression in iPSCs/ESCs were lower than that in MEF, its expression on Day 3 during reprogramming were suppressed to a level significantly lower than those in iPSCs and ESCs (Fig. 6a). This over down-regulation of *Hif1a* was resulted from the exogenous expression of *Oct4, Klf4*, and *Sox2* (Fig. 6a). Considering the interaction between LSD1 and these transcriptional factors (Fig. 3d), it was reasonable to proposed that Oct4, Klf4, and Sox2 induced H3K4 demethylation on *Hif1a* gene locus with the help from LSD1 and subsequently decreased *Hif1a* expression. When LSD1 was inhibited, such H3K4 demethylation was impaired and *Hif1a* expression was reversed.

To test the hypothesis above, ChIP-qPCR was used to determine the binding of OKS and LSD1 to *Hif1a* gene locus and to explore the changes on H3K4 methylation in the same locus. As indicated in Fig. 6b, Oct4, Klf4, Sox2 and LSD1 bound to *Hif1a* gene locus on Day 3 during reprogramming. In addition, ChIP-qPCR with antibodies against H3K4me2 and H3K4me3 suggested that the H3K4 methylation level on *Hif1a* gene locus decreased after OKS delivery, and could be reversed partially with T20 treatment (Fig. 6c,d). Therefore, Oct4, Klf4, and Sox2 bind to *Hif1a* gene locus and regulate related H3K4 methylation with the help from LSD1.

Then the contributions of *Hif1a* expression modulation and metabolic switch to T20’s functions during reprogramming were determined. As expected, an shRNA against *Hif1a* counteracted with T20 in regulating expression of representative genes in OX and glycolysis pathways (Fig. 6e), and reduced the ability of T20 to promote iPSCs generation (Fig. 6f). Additionally, the oxidative phosphorylation inhibitor, rotenone, increased reprogramming efficiency, which could be blocked by LSD1 over-expression at least partially (Fig. 6f). These observations further supported the conclusion that LSD1 inhibition assists the metabolic switch from oxidative to glycolytic by regulating *Hif1a* expression.

### T20 also promotes pre-iPSCs conversion by assisting metabolic switch

In addition, when analyzing current RNA-seq together with previous studies on pre-iPSCs, iPSCs, and ESCs^36^,^37^,^38^, LSD1 inhibition seemed to promote the conversion from pre-iPSCs to iPSCs (Supplemental Tables S1 and S3). Thus two already established pre-iPSCs lines, pre-2-2 and pre-3^39^, was used to determine the effects of T20 on the Vitamin C (Vc)-induced conversion from pre-iPSCs to iPSCs.

**Figure 7:**
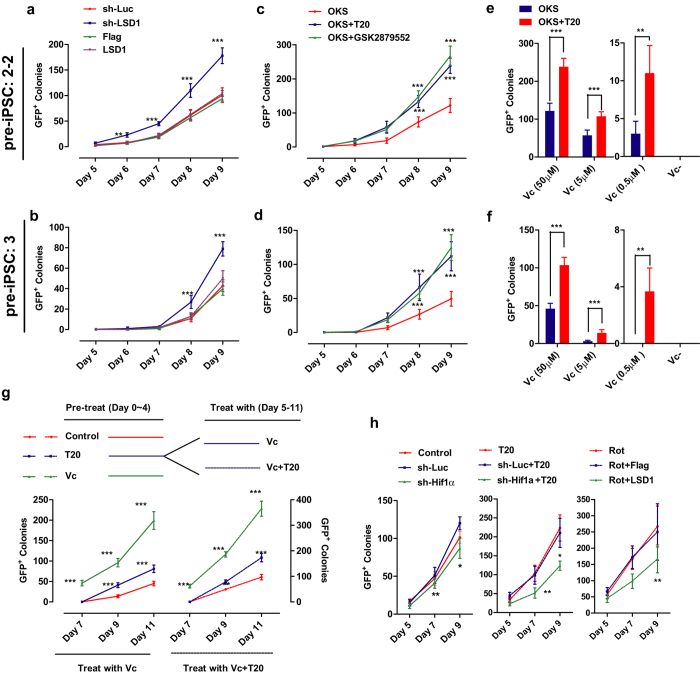
T20 promotes pre-iPSCs conversion by facilitating metabolic switch. Two pre-iPSCs cell lines, pre-2-2 and pre-3, were converted to iPSCs with Vc and indicated treatments. Numbers of GFP^+^ colonies were counted. (**a,b**) Retrovirus encoding sh-LSD1 and LSD1 were used for the conversion of pre-2-2 ((**a**), n = 6) and pre-3 ((**b**), n = 6). (**c,d**) T20 and GSK2879552 (2 μM) were used for the conversion of pre-2-2 ((**c**), n = 6) and pre-3 ((**d**), n = 6). (**e,f**) The effects of T20 on pre-iPSCs conversion were determined in pre-2-2 ((**e**), n = 6) and pre-3 ((**f**), n = 6) with reduced Vc concentrations. (**g**) Pre-2-2 were pre-treated with PBS (control), Vc and T20 from Day 0 to Day 4, and then treated with Vc or Vc+T20 from day 5 to Day 11. GFP^+^ colonies were counted. (**h**) Retrovirus encoding sh-Luc or sh-Hif1α was used with or without T20, and Rot was used with retrovirus encoding Flag or LSD1 during iPSCs generation. GFP^+^ colonies were counted.Two-way ANOVA with Bonferroni’s test as a post-hoc test was used.

As expected, both shRNA against *Lsd1* and treatment with T20 or GSK2879552 promoted the conversion by about two folds (Fig. 7a–d). To determine potential interaction between Vc and T20, the concentrations of Vc in the medium were reduced to different levels. Although T20 could not convert pre-iPSCs to iPSCs without Vc, it promoted these conversions significantly even at a much lower concentration (0.5 μM) of Vc (Fig. 7e,f). In addition, pre-treating pre-iPSCs with T20 for 4 days increased the following conversions no matter whether the cells were continued to be treated with Vc alone or with both Vc and T20 on Day 5 to 11 (Fig. 7g). Thus the beneficial roles of T20 on this conversion were independent from the functions of Vc.

In addition, the beneficial roles of T20 on the metabolic switch were also observed during the conversion of pre-iPSCs. shRNA against *Hif1a* significantly inhibited the beneficial roles of T20, while *Lsd1* over-expression also impaired rotenone-increased conversion efficiency (Fig. 7h).

## Discussion

The current study demonstrated the two mechanisms employed by LSD1-inhibition to promote iPSCs generation at early stage of reprogramming. On the one hand, LSD1 inhibition increases retrovirus-delivered exogenous expression of *Oct4, Klf4*, and *Sox2* by preventing related H3K4 demethylation. The increased exogenous expression of *Oct4, Klf4*, and *Sox2* contribute to the T20-faciliated reprogramming at least partially. On the other hand, because of the interactions between LSD1 and Oct4, Klf4 and Sox2, LSD1 inhibition impairs OKS-induced H3K4 demethylation on genes with relative high expression and H3K9 demethylation on genes with relative low expression (Supplemental Table S1). As indicated by the RNA-seq analysis, LSD1 inhibition partially impaired OKS-induced expression changes, like the expression changes of genes in OX and glycolysis pathways. Thus, although the two mechanisms seem to be controversial from each other, they both promote reprogramming and account for the beneficial roles of LSD1 inhibition.

Since LSD1 inhibition also promotes reprogramming even in a virus-free system, and LSD1 inhibition increases iPSCs generation by impairing some OKS-induced expression changes, some OKS-induced expression changes may be unfavorable for iPSCs generation. The typical unfavorable expression changes identified in the current studies were those of genes in OX and glycolysis pathways. Although cells transit from oxidative to glycolytic metabolism during iPSCs generation^33^, genes in both OX and glycolysis pathways were up-regulated during reprogramming when analyzing previous studies on pre-iPSCs, iPSCs, and ESCs^36^,^37^,^38^ (Supplemental Tables S1 and S3). OKS over up-regulated genes in OX pathway, but down-regulated genes in glycolysis pathway at early stage of reprogramming, which subsequently impaired metabolic switch. Inhibiting LSD1 partially reversed these expression changes, facilitated metabolic switch, and subsequently contributed to the beneficial roles of T20.

Then the question was to determine what is the percentage of such unfavorable expression changes in OKS-induced expression changes. Our hypothesis was that more than 50% OKS-induced expression changes was unfavorable. After analyzing current RNA-seq data together with previous studies on pre-iPSCs, iPSCs, and ESCs^36^,^37^,^38^ (Supplemental Tables S1 and S3), 2208 genes whose expression changed by more than 2 folds after OKS delivery in current RNA-seq were used for further analysis. Three expression ratios, OKS vs MEF, iPSC&ESC vs MEF, and pre-iPSC vs iPSC&ESC, were used to define “consistent”, “un-necessary”, “opposite”, and “over-regulated” changes induced by OKS (Supplemental Fig. S7). 693 genes (31%) had OKS-induced changes consistent with those from MEFs to iPSCs/ESCs, 1144 genes (52%) had opposite or un-necessary changes, and 203 genes (9%) seemed to be over-regulated (Supplemental Table S1 and Fig. S7). If considering opposite, un-necessary, and over-regulated changes as unfavorable changes, majority (61%) of expression changes induced by OKS were unfavorable for reprogramming.

The 61% above was calculated on Day 3 in current reprogramming system, another microarray datasets of a 21-day reprogramming system was analyzed^38^. Genes with unfavorable regulations by OKS decreased during at late stage of reprogramming (Supplemental Fig. S7). In addition, when calculating the percentages of unfavorable expression changes at different time points in the 21-day reprogramming system, the percentages peaked on Day 5 and gradually decreased (Supplemental Fig. S7), suggesting a significant portion of expression changes induced by OKS were unfavorable especially at early stage. Therefore, LSD1 inhibition might promote reprogramming by reversing some of these unfavorable changes.

The “unfavorable” hypothesis above still had additional challenges. These unfavorable changes might be a necessary stage for reprogramming and might be not really “unfavorable”. In addition, RNA-seq and microarray data were generated from large population of cells during reprogramming and were contaminated with cells that were not on their way to iPSCs. Thus further testing on this hypothesis should be based on addition single-cell tracing and expression profile analysis.

In addition, another recent report on autophagy and mechanistic target of rapamycin complex 1 (*mTORC1*) supported current hypothesis from another aspect^40^. OKS down-regulated *mTORC1* expression, but up-regulated genes involved in the elongation and expansion of autophagy like *Atg5, Atg7, Atg4b* and *Atg16l*. T20 might also facilitate reprogramming by reversing these gene expression and suppressing autophagy.

## Methods

### Animal Studies

Our studies were performed in accordance with the guidelines for the Care and Use of Laboratory Animals of the National Institutes of Health. The protocols were approved by the Committee on the Ethics of Animal Experiments of the Guangzhou Institutes of Biomedicine and Health. All efforts were made to minimize the number of animal and the animal discomfort.

### Generation of iPSCs

MEFs were derived from 13.5-day mouse embryos carrying the *Oct4*-GFP transgenic allele^41^. These MEFs were maintained in DMEM (Gibco) supplemented with 10% FBS (Gibco), nonessential amino acids (NEAA, Gibco), penicillin/streptomycin (P/S, Thermo), and GlutaMAX (Gibco). Retrovirus was produced with Plat-E cells and pMXs-based retroviral vectors as previous reported except calcium phosphate transfection protocol was used^42^. MEFs within two passages were split into twelve-well plate (1.5 × 10^4^ cells/well). After adding polybrene to 4 μg/ml, the viral supernatant was used for infection. *Oct4, Klf4, c-Myc* and *Sox2* or *Oct4, Klf4* and *Sox2* were introduced into cells twice on Day 0 and Day 1 respectively, while mES or mES-Vc [high glucose DMEM (Hyclone), NEAA, P/S, GlutaMAX, leukemia inhibitory factor (LIF), β-mercaptoethanol, pyruvate, and 10% FBS without or with Vc (Sigma, 49752)] was used on Day 2. Medium was replaced with freshly prepared medium every day. If inhibitor, like GSK2879552 or T20, was used, it was also added to the fresh medium from the stock solution every day. iPSC colonies were counted or picked according to their Oct4-GFP expression and ES-like morphology, on Day 12–14. R1 ESCs and iPSCs were cultured on MEF feeder cells in Knockout DMEM supplemented with Knockout Serum Replacement (KSR, Gibco), LIF, PD0325901, CHIR-99021, NEAA, P/S, GlutaMAX, and β-mercaptoethanol.

The CiPSCs were generated as previously reported^16^. Briefly, MEFs were seeded at a density of 50,000 cells per well of a 6-well plate. On Day 0, the medium was replaced with KnockOut DMEM containing 10% KSR, 10% FBS, NEAA, GlutaMAX, P/S, 100 ng/mL bFGF, 500 μM VPA, 20 μM CHIR99021, 10 μM 616452, 20 μM Forskolin and 1 μM TTNPB. The medium was changed every 4 days. On Day 12, these cells were replated at a density of 30,000 cells per well of a 6-well plate. The medium was replaced with KnockOut DMEM containing 10% KSR, 10% FBS, NEAA, GlutaMAX, P/S, 20 ng/mL bFGF, 500 μM VPA, 10 μM CHIR99021, 10 μM 616452, 10 μM Forskolin and 1 μM TTNPB. DZNep was added to the cell cultures on day 16. On day 28, the medium was replaced with KnockOut DMEM containing 10% KSR, 10% FBS, NEAA, GlutaMAX, P/S, 3 μM CHIR99021 and 1 μM PD0325901 until the AP staining on Day 36. 20 μM Tranylcypromine was used during indicated time periods.

HFFs were cultured in DMEM with 10% FBS, NEAA, GlutaMAX and P/S. 1 × 104 HFFs were plated into every well of 24-well plate. On Day 0 and 1, HFFs were incubated with retrovirus-containing supernatant with 4 μg/ml polybrene for 12 hours followed with a 12-hour rest. On day 2, medium was changed to DMEM with 20% defined FBS, and bFGF (Shenzhen Symmix Industry). On Day 4, Vc and VPA (1 mM; Merck, Germany) was added to the medium. Conditioned medium, the supernatant collected and filtered 12 hours after MEF feeder cells were cultured in current medium, was used from Day 10 until the AP staining on Day 24.

### Cell line characterization

Characterization of iPSCs were done as previously reported^42^. The primary antibodies used were: goat anti-NANOG (R&D Systems, AF2729, 1:500), and mouse anti-SSEA-1 (R&D Systems, FAB2155A, 1:500). Appropriate Alexa-568-conjugated secondary antibodies were purchased from Invitrogen (A10037, A10042 and A10057, 1:500). For karyotypes analysis, demecolcine (50 μg /ml, Dahui Biotech) was added to cells for 1 h before addition preparation and metastages were analyzed on an Olympus BX51 microscope.

For immunoblotting, mouse anti-β-actin (Sigma, A5316-clone-AC-74, 1:2000), rabbit anti-GAPDH (Abcam, ab37168, 1:1000), rabbit anti-LSD1 (Abcam, ab17721, 1:1000), mouse-anti-H3K4me2 (Abcam, ab7766, 1:2000), H3K4me3 (Abcam, ab8580, 1:2000), H3K9me2 (Abcam, ab1220, 1:2000), H3K9me3 (Abcam, ab8898, 1:2000), and H3 (Abcam, ab1791, 1:5000) were used as primary antibodies. Appropriate HRP-conjugated secondary antibodies were purchased from Promega (W4011 and W4021, 1:500). The gels used for different antibodies were run under the same experimental conditions. Representative images were cropped from the original images with no modification on the relative intensities. The full length blots were provided in Supplementary Figure S8.

### AP staining and TUNEL staining

Cells were fixed with 4% paraformaldehyde in PBS, incubated at room temperature for 2 min, then washed twice with 0.5 ml of TBST. Freshly prepared AP staining solution (10 ul/ml nitrobluetetrazolium, 2 ul/ml5-bromo-4-chloro-3-indolyl phosphatein 100 mM Tris-HCl, pH 9.5, 100 mM NaCl, 50 mM MgCl_2_) was added, and plates were incubated in the dark at room temperature for 15 min, then rinsed with PBS. Apoptotic cell death was analyzed by the TUNEL assay using the *in situ* Cell Death Detection Kit, TMR red (Roche).

### Quantitative RT-PCR (qPCR)

Total RNA was extracted from cells by using TRIzol (Invitrogen) and 5 μg RNA was used to synthesize cDNA with ReverTra Ace^®^ (Toyobo) and oligo-dT (Takara) according to the manufacturer’s instructions. Transcript levels of genes were determined by using Premix Ex Taq™ (Takara) and analyzed with CFX-96 Real Time system (Bio-Rad). The primers were listed in Table S5.

### RNA-seq

RNA was extracted from cells using TRIzol reagent (Invitrogen). Illumina mRNA-seq libraries were prepared for each RNA sample by using the TruSeq RNA Sample Preparation Kit v2 (Illumina) before sequencing on an Illumina MiSeq instrument with the MiSeq Reagent Kit (Illumina).

Obtained RNA-Seq reads were processed by RSEM (RNA-Seq by Expectation-Maximization) to estimate transcript abundances. Reads were aligned to the (Ensembl v67) transcriptome and the number of reads associated with a given transcript was used to estimate that transcript’s abundance in TPM (transcripts per million).

### ChIP-qPCR

Chromatin from cells was fragmented to a size range of 200–500 bases with a Sonicator. Solubilized chromatin was immunoprecipitated with antibody against H3K4me2 (Abcam, ab7766, 1:1000), H3K4me3 (Abcam, ab8580, 1:1000). Antibody–chromatin complexes were pulled-down with protein A/G (Invitrogen), washed and then eluted. After cross-link reversal and Proteinase K treatment, immunoprecipitated DNA was extracted with phenol-chloroform, ethanol precipitated, and treated with RNase. ChIP DNA was quantified using PicoGreen. For ChIP-qPCR, primer sequences for qPCR tiling primers are listed in Table S5. qPCR was performed on CFX-96 Real Time system (Bio-Rad)CFX-96 Real Time system (Bio-Rad) with Premix Ex Taq™ (Takara).

### Bioenergetic profiling

Seahorse XF24 extra-cellular flux analyzer (Seahorse Bioscience) was used to assess cellular energy metabolism following the instruction of instruments. Briefly, the instrument quantified mitochondrial respiration (oxygen consumption rate, OCR) and glycolysis to lactic acid (extracellular acidification rate, ECAR). Four mitochondrial inhibitors (all from Sigma) were used in succession. After 30-min basal measurements,1 μM oligomycin was added to inhibit OXPHOS. 1 μM FCCP was injected into the wells to induce the collapse of the mitochondrial membrane potential after the measurement at 60 min. 1 μM rotenone and 1 μM antimycin A were simultaneously injected to completely inhibit mitochondrial respiration after the measurement at 90 min.

### Statistical methods

Experiments were repeated at least five times (n > = 5) with the exception of RNA-seq analysis. Data were analyzed and compared by two-tailed t-test, one-way ANOVA with Dunnett’s test as a post-hoc, or two-way ANOVA with Bonferroni’s test as a post-hoc test with GraphPad Prism 5.0. Error bars and “n” represented standard deviations (stand error if mentioned) and the number of independent experiments. “*”, “**”, and “***” represented significant differences (p < 0.05), (p < 0.01), and (p < 0.001) from indicated control groups, respectively.

## Additional Information

**Accession code**: RNA-seq data were deposited in the Gene expression omnibus under accession number: GSE61694.

**How to cite this article**: Sun, H. *et al*. Lysine-specific histone demethylase 1 inhibition promotes reprogramming by facilitating the expression of exogenous transcriptional factors and metabolic switch. *Sci. Rep.* **6**, 30903; doi: 10.1038/srep30903 (2016).

## Supplementary Material

Supplementary Figures and Table Legends

Supplementary Table S1

Supplementary Table S2

Supplementary Table S3

Supplementary Table S4

Supplementary Table S5
