# Supplementary material for: Lysine-specific histone demethylase 1 inhibition promotes reprogramming by facilitating the expression of exogenous transcriptional factors and metabolic switch
Source: Sci Rep. 2016 Aug 2;6:30903. doi: 10.1038/srep30903 (PMC4969595; doi:10.1038/srep30903)
Supplement: Supplementary Figures and Table Legends [file srep30903-s1.pdf]

Supplementary Information:

Lysine-specific histone demethylase 1 inhibition promotes reprogramming by facilitating the expression of exogenous transcriptional factors and metabolic switch

Hao Sun[1,2], Lining Liang[1], Yuan Li[1], Chengqian Feng[1], Lingyu Li[1], Yixin Zhang[1], Songwei He[1], Duanqing Pei[1], Yunqian Guo[2,*], & Hui Zheng[1,*]

[1]CAS Key Laboratory of Regenerative Biology, Guangdong Provincial Key Laboratory of Stem Cell and Regenerative Medicine, Guangzhou Institutes of Biomedicine and Health, Chinese Academy of Sciences, Guangzhou 510530, China.

[2]College of Biological Sciences and Technology, Beijing Forestry University, Beijing 100083, China

[*]Correspondence should be addressed to Guo Yunqian or Zheng Hui, Guangzhou Institutes of Biomedicine and Health, A-131, 190 Kaiyuan Ave. Science City, Guangzhou, 510530, China. Tel: +86-20-32015334. Fax: +86-20-32015231. E-mail: zheng_hui@gibh.ac.cn.

Supplementary Figures

Figure S1

T20 promotes reprogramming with OKMS

(a-b) Retrovirus encoding sh-LSD1, LSD1 and K661A were used to infect MEFs for 3 days. *Lsd1* expression was determined by immunoblotting (a, n=5) and qPCR (b, n=5).

(c) The ability of sh-LSD1 to suppress *Lsd1* expression was determined during OKS-induced reprogramming by performing qPCR on Day 3, 6, 9, and 12 (n=5). Representative immunoblotting results on Day 9 were also provided.

(d-f) Different concentrations of tranylcypromine were used in reprogramming with OKMS and 200 μM tranylcypromine (T200) was determined to be optimal (d). Retrovirus encoding sh-LSD1*,* LSD1, and K661A were used in different combinations with or without T20 during OKMS reprogramming (e). T200 was used in different time periods during reprogramming (f). Numbers of GFP^+ colonies were summarized (n=6).

(g-h) Different concentrations of another two LSD1 inhibitors, phenelzine and GSK2879552, were used in reprogramming with OKS (g) or OKMS (h). Numbers of GFP^+ colonies were summarized (n=6).

(i) 100 μM Phenelzine and 2 μM GSK2879552 were used in reprogramming with OKS in a time-dependent manner. GFP^+ colonies were counted to indicate the reprogramming efficiencies (n=6).

Two-way ANOVA with Bonferroni's test as a post-hoc was used for (c).

One-way ANOVA with Dunnett's test was used for (d-i).

The gels used for different antibodies were run under the same experimental conditions (a and c). Representative images were cropped from the original images

with no modification on the relative intensities. The full length blots were provided in Supplementary Figure S8.

Associated with Figure 1.

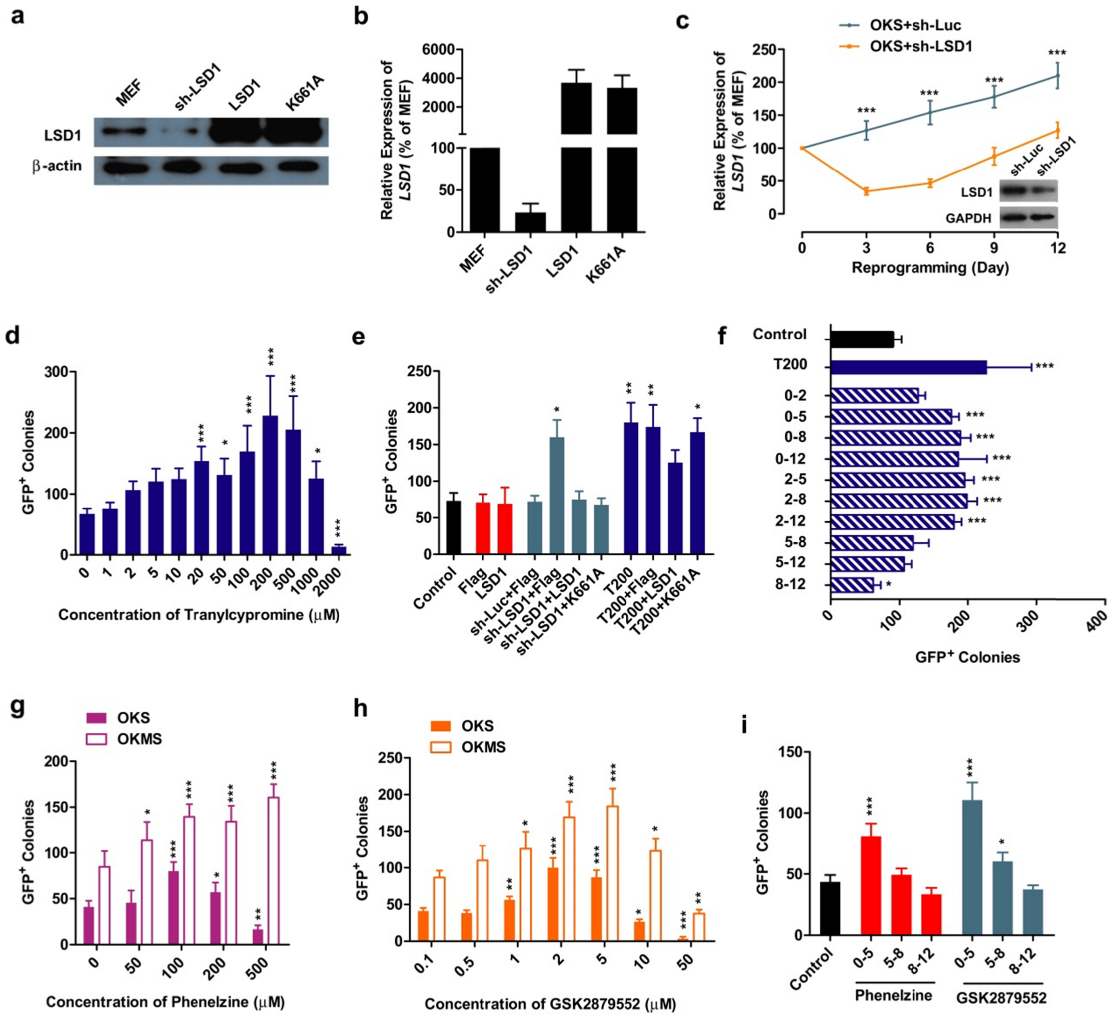

Figure S2

Characterization of iPSCs generated with OKS and T20

GFP^+ colonies generated with OKS and T20 were picked out, cultured and characterized. MEFs and R1 ESCs were used as controls.

(a) Endogenous expression of the four factors (*Oct4*, *Klf4*, *c-Myc* and *Sox2*) were determined by qPCR (n=5).

(b) Karyotypes of the cells were normal.

(c) DNA demethylation on the promoter of *Oct4* and *Nanog* was observed.

(d) Fluorescence staining of pluripotency markers (Nanog and SSEA1) were positive (n=5).

(e) The cells were able to form chimeric mice with germ line transmission.

Associated with Figure 1.

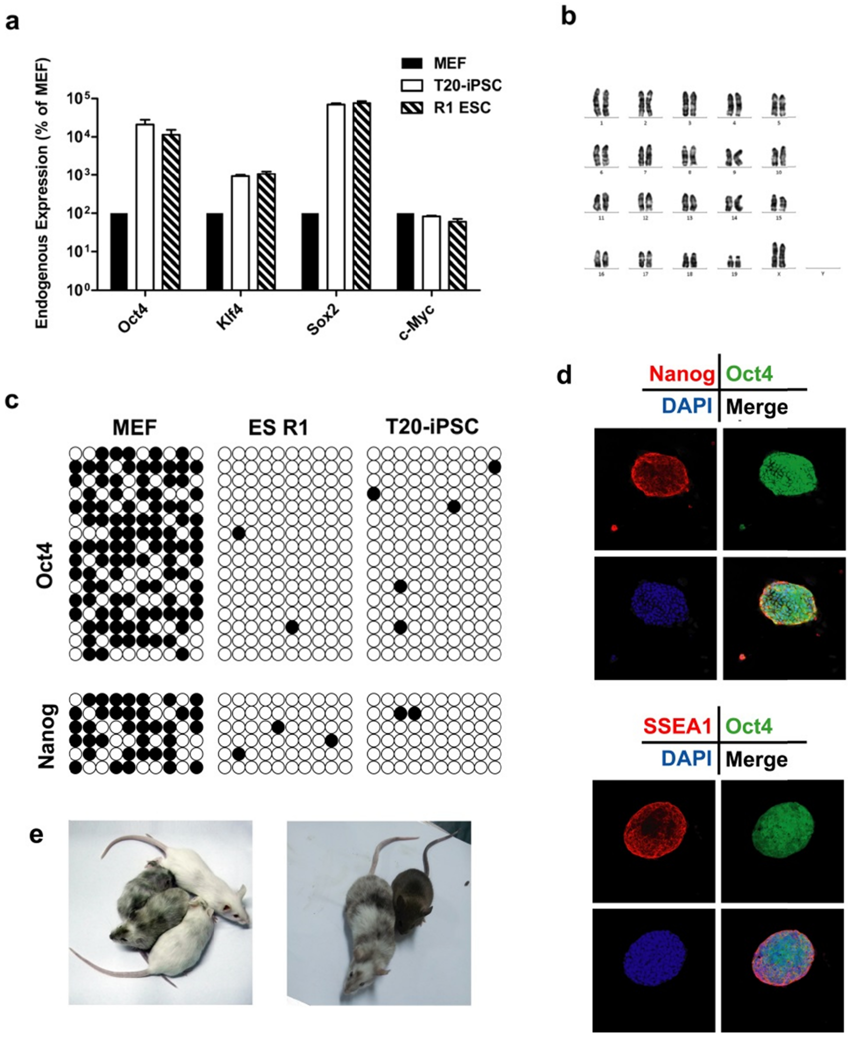
a
MEF
T20-iPSC
R1 ESC
Endogenous Expression (% of MEF)
Oct4
Klf4
Sox2
c-Myc
b
c
MEF
ES R1
T20-iPSC
Oct4
Nanog
d
Nanog
Oct4
DAPI
Merge
SSEA1
Oct4
DAPI
Merge
e

Figure S3

T20 does not affect cell cycle and DNA methylation

OKS reprogramming was performed without (OKS) or with 20 μM tranylcypromine (OKS+T20). 500 μM tranylcypromine (OKS+T500) was also used.

(a) Cell amounts were recorded during reprogramming (n=5).

(b) Distributions of cells into different phases of cell cycle were determined by flow cytometry on Day 3 and Day 6 (n=5).

(c) Apoptosis rates were determined by TUNEL assay on Day 3, 6, and 9 (n=5).

(d-e) Global DNA methylation was assessed by dot-blot with antibody against 5mC on Day 3 (e, n=5) and with HPLC (f, n=5).

(f) Expression of *Dnmt1* and *p53* were determined by qPCR on Day3 (n=5).

Two-way ANOVA with Bonferroni's test was used for (a).

One-way ANOVA with Dunnett's test was used for (b and e).

Two-tailed student t-test was used for (c and f).

Associated with Figure 2.

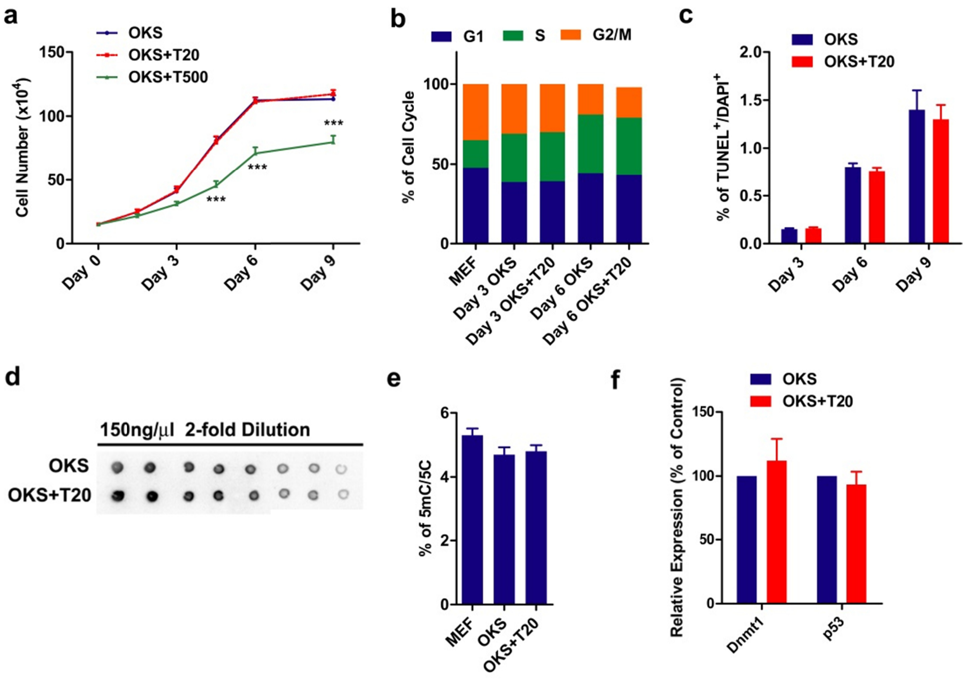

Figure S4

H3K4 and H3K9 methylation during reprogramming

(a) The effects of retrovirus encoding sh-Lsd1 and sh-Dpy30 on related gene expression (*Eset* and *Dpy30*) were determined by qPCR in MEFs 3 days after delivery (n=5).

(b-c) H3K4me2/3 and H3K9me2/3 were determined by immunoblotting in MEFs on Day 3 during reprogramming with/without T20. Histone 3 served as internal control. Sample images and average data were listed in (b, n=5) and (c, n=5) respectively. One-way ANOVA with Dunnett's test was used for (c).

The gels used for different antibodies were run under the same experimental conditions (b). Representative images were cropped from the original images with no modification on the relative intensities. The full length blots were provided in Supplementary Figure S8.

Associated with Figure 2.

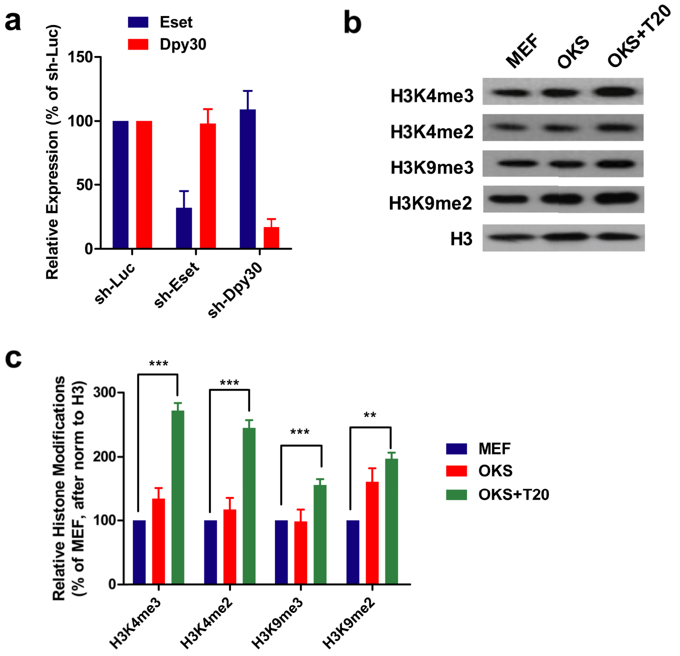

Figure S5

Genes expression profiles in current RNA-seq

(a-b) Log_2 values of gene expression in MEF and OKS groups (a) or in MEF and OKS+T20 groups (b) were plotted against each other. Red lines were used to distinguish the genes with expression changes more than 5 folds.

(c) 9399 genes in current RNA-seq were divided into 14 groups depending on the ratios of OKS to MEFs. The averages of gene expression in MEF were plotted. (Error bars represented s.e.m.)

One-way ANOVA with Dunnett's test as a post-hoc was used.

Associated with Figure 3.

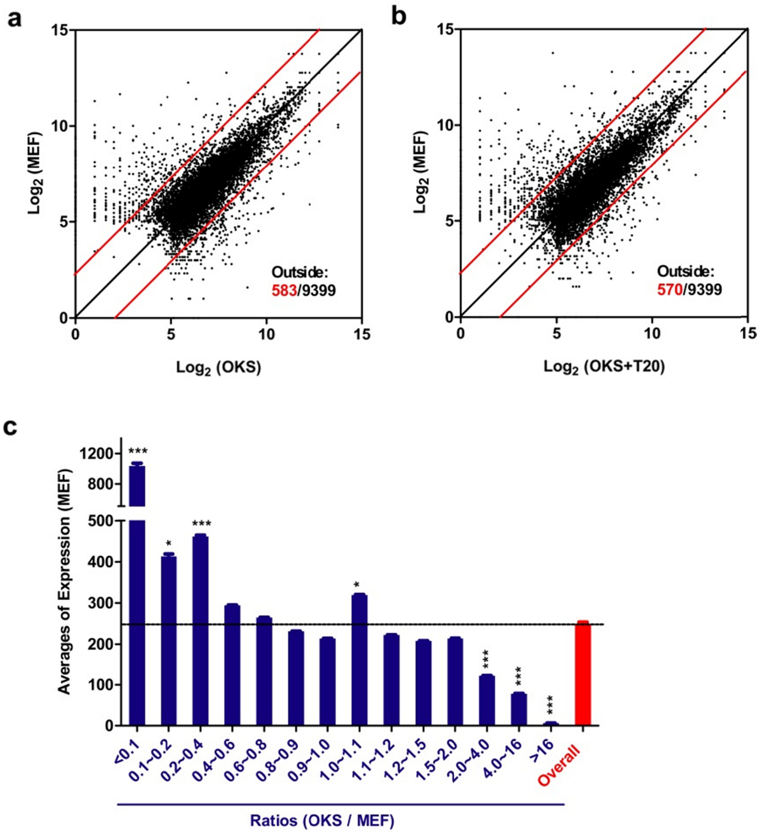

Figure S6

GSK2879552 and sh-LSD1 facilitates metabolic switch

(a-b) qPCR results of *Nrf1*, five representative genes in OX pathway (a), *Hif1a*, and five representative genes in glycolysis pathway (b) in MEFs, OKS+sh-Luc, OKS+sh-LSD1, and OKS+sh-Luc+GSK2879552 groups were listed (n=5).

(c-d) Seahorse XF24 extra-cellular flux analyzer was used to assess cellular energy metabolism on Day 3 during reprogramming. GSK2879552 and shRNAs were added as indicated. OCR and ECAR results were presented in (c) and (d) respectively (n=5).

One-way ANOVA with Dunnett's test as a post-hoc was used (a-b).

Associated with Figure 5.

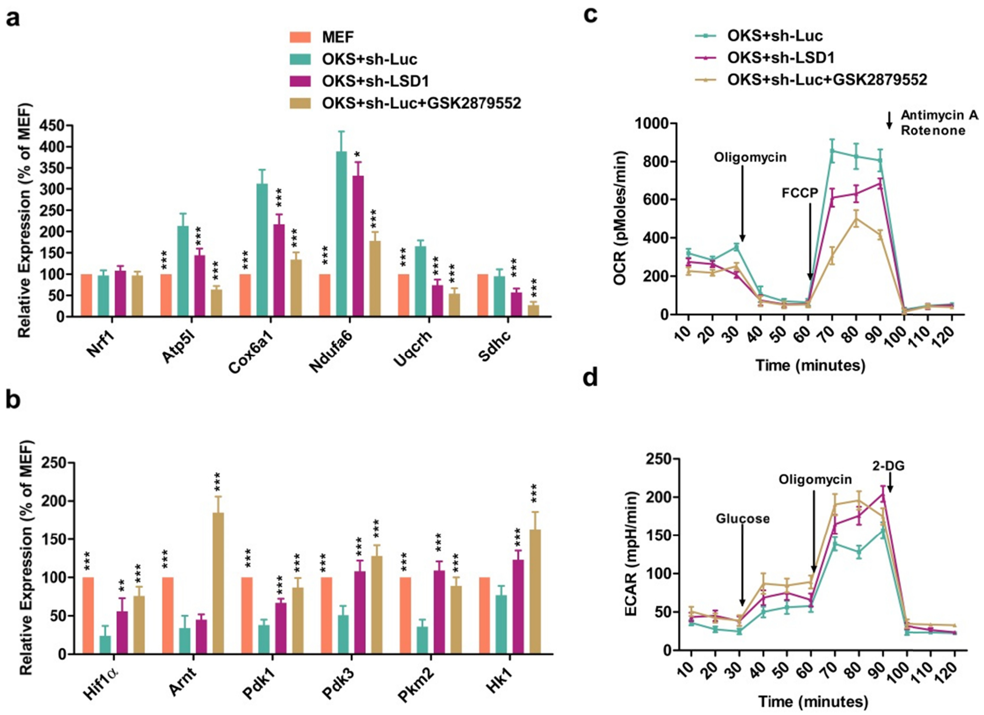

Figure S7

“Unfavorable ” expression change models for reprogramming

(a) OKS-induced significant expression changes (> 2 folds) were classified with the ratios of gene expression between iPSCs & ESCs and MEFs (67% and 150%) and those between pre-iPSCs and iPSCs & ESCs. (Error bars represented S.E.M.)

(b) Genes significant up-regulated by OKS were classified into four groups as in (a). Averages of their expression (Log$_2$ values) during a 22-day reprogramming were plotted. (Error bars represented S.E.M.)

(c) Similar classification were performed as in (a) during a 22-day reprogramming, the overall percentages of genes classified as over-regulated, un-necessary and opposite were plotted.

Associated with Discussion.

a

OKS/ MEF	iPSC & ESC/ MEF	pre-iPSC/ iPSC	Gene No. (Remark)	Ave. of Log$_2$ (OKS+T20/OKS)
>200%	>150%	<150%	238 (Consistent)	-0.25±0.06
		>150%	121 (Over-regulated)	0.13±0.07
	67~150%		403 (Un-necessary)	-0.26±0.05
	<67%		253 (Opposite)	-0.04±0.04
<50%	>150%		154 (Opposite)	0.64±0.06
	67~150%		334 (Un-necessary)	0.75±0.05
	<67%	<67%	82 (Over-regulated)	0.26±0.07
		>67%	455 (Consistent)	0.50±0.04

*168 genes were not analyzed because of data missing

b

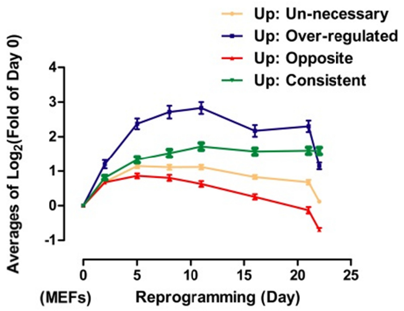

c

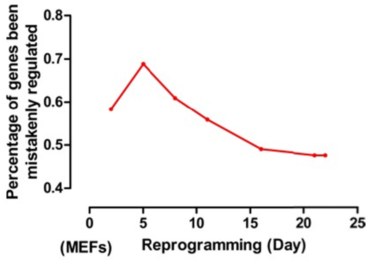

Figure S8

Full images of cropped immunoblotting results

(a) Full images of cropped immunoblotting results in Figure 1a. Protein levels of LSD1 were placed in up-panel.

(b) Full images of cropped immunoblotting results in Figure 3a. Co-IP for different transcription factors were listed.

(c) Full images of cropped immunoblotting results in Supplementary Figure S1a. Protein levels of LSD1 were placed in up-panel.

(d) Full images of cropped immunoblotting results in Supplementary Figure S1c. Protein levels of LSD1 were placed in up-panel.

(e) Full images of cropped immunoblotting results in Supplementary Figure S4b. Histone modifications were listed.

a for Figure 1a

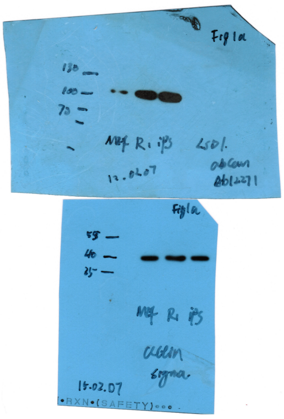

b for Figure 3a

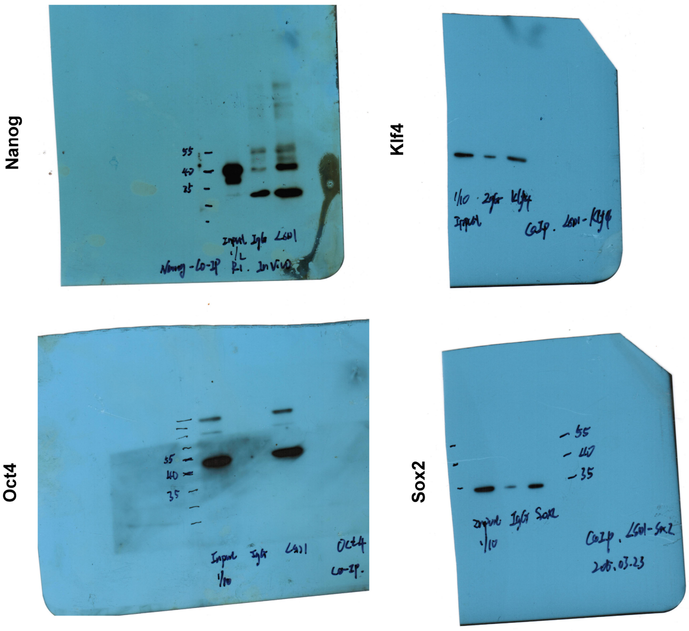

c for Figure S1a

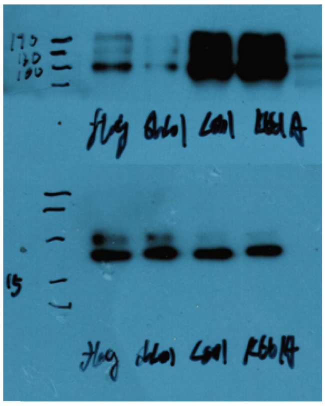

d for Figure S1c

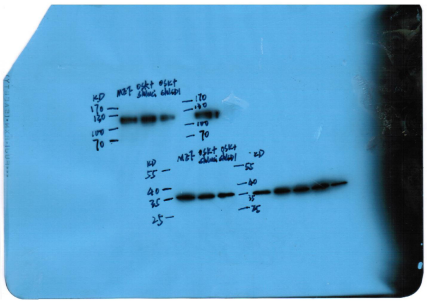

e for Figure S4b

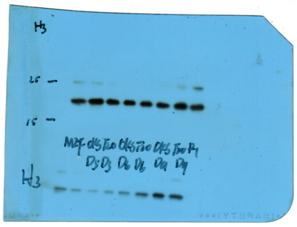

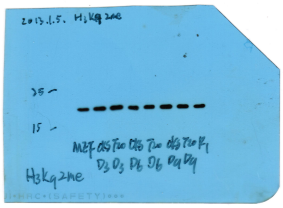

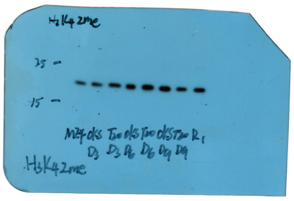

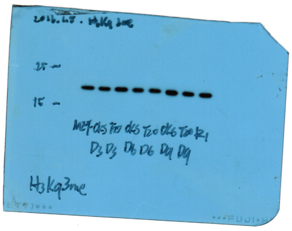

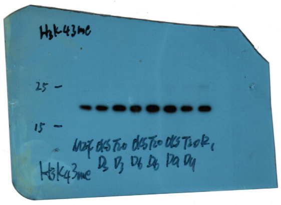

Supplementary Tables

Table S1

Expression changes of 9399 genes identified in current manuscript together with those in another three microarray datasets

Expression changes of 9399 detectable genes in current RNA-seq were analyzed together with data from GSE14012, GSE10871, and GSE21757 to provide an overall view for these genes' expression during reprogramming and in pre-iPSCs and ESCs. Normalized readings in MEFs from RNA-seq and microarrays were provided in the Table. The readings in pre-iPSCs, iPSCs, ESCs and during a 22-day reprogramming were provided as ratios to those in MEFs.

2208 genes whose expression were modulated by OKS over 2-folds were marked "Yes" in column "Z". Associated with Figure 3&4.

Table S2

Gene ontology analysis of genes with significant expression differences

GO analysis of genes with ratios of OKS+T20 to OKS over 200% in (sheet 1, "Increased GO") and below 50% in (sheet 2, "Decreased GO"). Genes analyzed in the second sheet were also analyzed for KEGG annotation (sheet3, "Decreased KEGG"). Associated with Figure 4.

Table S3

The expression of genes in oxidative phosphorylation and glycolysis pathway

116 genes in OX and 60 genes in glycolysis pathways were picked out from Table S1 for collective analysis. Associated with Figure 4.

Table S4

Binding motif analysis summary on genes in oxidative phosphorylation and glycolysis pathway

Genes with expression changed more than two fold during T20 treatment were subjected for Pscan analysis. The binding motifs with p-values smaller than 1.0e-04 in both up-regulated (column "A-C") and down-regulated (column "D-F") genes were listed on the top and considered as set "First 2".116 genes in OX and 60 genes in glycolysis pathways were also used for Pscan analysis to identify possible enriched binding motifs. In OX pathway (column "I-L"), binding motifs with p-values smaller than 1.0e-03 and been identified in set "First 2" were marked "Yes" in column "L". In glycolysis pathway (column "N-Q"), binding motifs with p-values smaller than 1.0E-02 and been identified in set "First 2" were marked "Yes" in column "Q".

Associated with Figure 5&6.

Table S5

Primers and sh-RNA sequences used in current manuscript.

As title.
